# MicroRNA 3928 Suppresses Glioblastoma through Downregulation of Several Oncogenes and Upregulation of p53

**DOI:** 10.3390/ijms23073930

**Published:** 2022-04-01

**Authors:** Elizabeth Q. X. Mulcahy, Ying Zhang, Rossymar R. Colόn, Shelby R. Cain, Myron K. Gibert, Collin J. Dube, Markus Hafner, Roger Abounader

**Affiliations:** 1 Department of Microbiology, Immunology & Cancer Biology, University of Virginia, Charlottesville, VA 22908, USA; qx3k@virginia.edu (E.Q.X.M.); yz5h@virginia.edu (Y.Z.); rr5fr@virginia.edu (R.R.C.); src7jv@virginia.edu (S.R.C.); mkg7x@virginia.edu (M.K.G.J.); cjd7ua@virginia.edu (C.J.D.); 2 National Institutes of Health (NIH), Bethesda, MD 20894, USA; markus.hafner@nih.gov; 3 Department of Neurology, University of Virginia, Charlottesville, VA 22908, USA; 4 University of Virginia Comprehensive Cancer Center, Charlottesville, VA 22908, USA

**Keywords:** miR-3928, glioblastoma, p53, MDM2, CD44, DDX3X, HMGA2, CCND1, BRAF

## Abstract

Glioblastoma (GBM) is the most frequent and lethal primary malignant brain tumor. Despite decades of research, therapeutic advances that significantly prolong life are non-existent. In recent years, microRNAs (miRNAs) have been a focus of study in the pathobiology of cancer because of their ability to simultaneously regulate multiple genes. The aim of this study was to determine the functional and mechanistic effects of miR-3928 in GBM both in vitro and in vivo. To the best of our knowledge, this is the first article investigating the role of miR-3928 in GBM. We measured endogenous miR-3928 expression levels in a panel of patient-derived GBM tissue samples and cell lines. We found that GBM tissue samples and cell lines express lower levels of miR-3928 than normal brain cortex and astrocytes, respectively. Therefore, we hypothesized that miR-3928 is a tumor suppressive microRNA. We verified this hypothesis by showing that exogenous expression of miR-3928 has a strong inhibitory effect on both cell growth and invasiveness of GBM cells. Stable ex vivo overexpression of miR-3928 in GBM cells led to a reduction in tumor size in nude mice xenografts. We identified many targets (MDM2, CD44, DDX3X, HMGA2, CCND1, BRAF, ATOH8, and BMI1) of miR-3928. Interestingly, inhibition of the oncogene MDM2 also led to an upregulation of wild-type p53 expression and phosphorylation. In conclusion, we find that miR-3928, through the downregulation of several oncogenes and upregulation and activation of wild-type p53, is a strong tumor suppressor in GBM. Furthermore, the fact that miR-3928 can target many important dysregulated proteins in GBM suggests it might be a “master” regulatory microRNA that could be therapeutically exploited.

## 1. Introduction

Mature microRNAs (miRNAs) are small noncoding RNAs that range in size from 18–22 nucleotides [1]. Since their discovery nearly 20 years ago, approximately 2000 unique microRNAs have been identified [2]. MicroRNAs regulate gene expression by targeting the mRNAs of many human genes. As a result, they play significant roles in the regulation of human physiology and pathobiology [3]. A main mechanism through which microRNAs modulate and regulate gene and protein expression is by binding to the 3′ untranslated region (3′ UTR) of the target mRNA, thereby promoting RNA degradation and/or inhibiting mRNA translation [4]. Since 100% binding complementarity is not necessary for the inhibition of target genes, single miRNAs can target multiple mRNAs.

In cancer, miRNAs are frequently deregulated, and their deregulation has been associated with various aspects of cancer pathobiology [3,5,6,7,8,9,10,11]. They can enhance or diminish malignancy by inhibiting the expression of tumor suppressors or oncogenes, respectively [9].

Glioblastoma (GBM) is the most common malignant brain tumor in adults, accounting for 52% of all primary malignant brain tumor cases [12]. Despite extensive research efforts, the outcomes of GBM patients remain poor [13,14]. Treatment for primary brain tumors such as GBM faces various challenges, including the simultaneous deregulation of multiple pathways in any given tumor [15]. GBM mutations lead to the deregulation of many key signaling pathways involving growth, proliferation, survival, and apoptosis [16].

Over the past decade, there has been increased interest in the regulatory role of miRNAs in GBM [7,14,17,18,19]. Because single miRNAs can act to simultaneously inhibit many genes, this unique property makes miRNAs attractive potential therapeutic molecules for GBM therapy [4,20,21,22,23].

To date, there are only four publications on the role and function of miR-3928 in cancer and no publications in GBM [24,25,26,27]. MiR-3928 was first shown to play an important role in the cellular response to ionizing radiation [28]. Because of its implication in cell cycle arrest, it was hypothesized to play a role in cancer. The first study on miR-3928 in cancer was done in osteosarcoma tissues and cells [25]. MiR-3928 was found to be down-regulated in osteosarcoma and overexpression was found to inhibit tumor growth through direct binding to ERBB3, CDK6, CDK13, and IL6R. These genes are involved in the processes of bone tissue growth, cell cycle regulation, and immune response.

Sequence alignments and bioinformatics analyses performed in our lab suggested that miR-3928 can theoretically target many important oncogenes in GBM, including MDM2, CD44, DDX3X, HMGA2, CCND1, BRAF, ATOH8, and BMI1.

The large number of potential miR-3928 targets and their involvement in a wide range of GBM signaling pathways prompted our investigation of the functional role of miR-3928 in GBM cell lines and animal models. Due to the many oncogenic targets of miR-3928, we hypothesized that it might play a significant role in the inhibition of GBM cell growth and invasion. We demonstrated that miR-3928 is down-regulated in GBM tumor tissue samples and GBM cell lines and that it strongly suppresses growth and invasiveness in a panel of GBM cell lines as well as in in vivo GBM xenografts. We show that miR-3928 exogenous expression leads to the degradation of multiple important oncogenes. It also leads to the upregulation and activation of wild type p53. Therefore, we consider miR-3928 a potential “master” regulatory miRNA that regulates GBM malignancy through the targeting of numerous genes relevant to GBM.

## 2. Results

### 2.1. miR-3928 Expression Is Downregulated in GBM Tumors and Cell Lines

**Figure 1:**
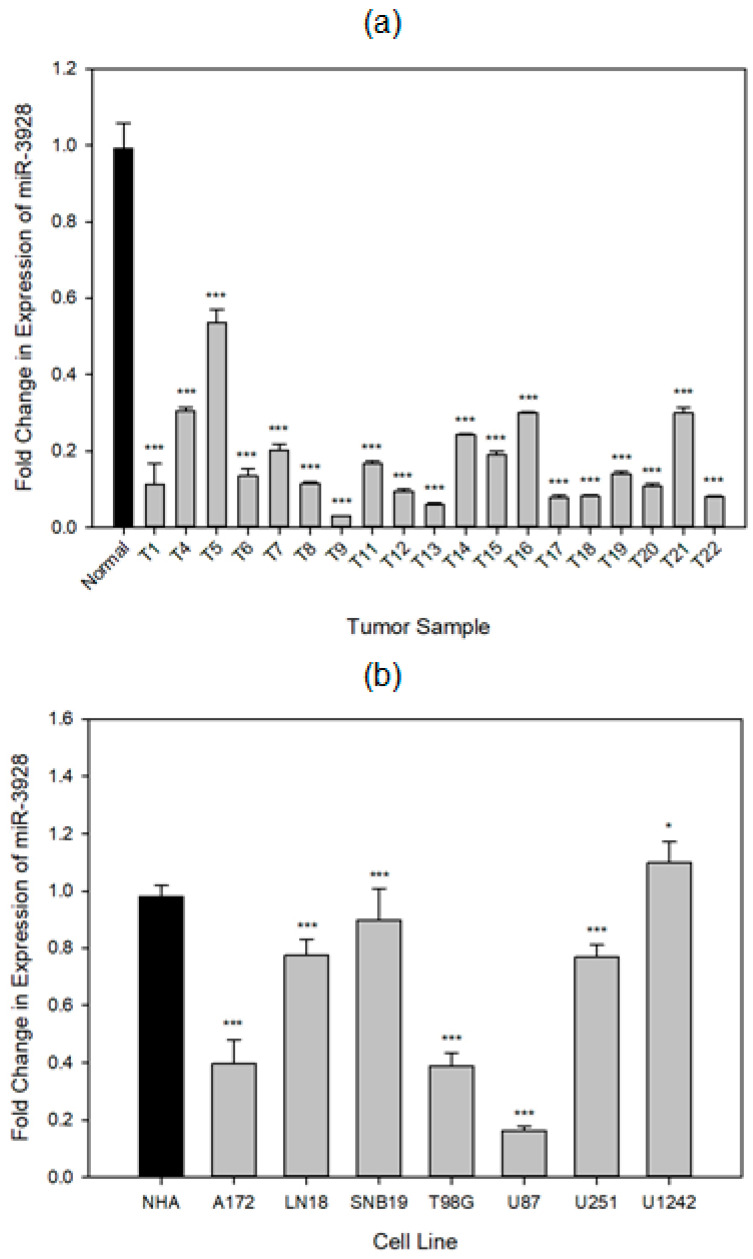
**miR-3928 expression is downregulated in GBM tumor and cell lines.** (**a**) The endogenous levels of miR-3928 were measured with RT-qPCR in a panel of GBM patient tissue samples. The values for each tissue sample are normalized to normal brain cerebral cortex and the housekeeping gene, RNU6B, U6B small nuclear RNA gene. miR-3928 was considered deregulated when the differences in mean expression was determined to be statistically significant (*p* < 0.05). The endogenous levels of miR-3928 are downregulated in all measured GBM tissue samples. (**b**) The endogenous levels of miR-3928 are measured through RT-qPCR in 7 commonly used GBM cell lines. The values are normalized to normal human astrocytes (NHA) and U6B. The endogenous levels of miR-3928 are downregulated in 6 of 7 GBM cell lines. All RT-qPCR samples were subsequently analyzed on an agarose gel to confirm binding specificity. Student’s *t*-tests have been performed to compare the qPCR cycle numbers. * = *p* ≤ 0.05, *** = *p* ≤ 0.001.

We first measured the endogenous levels of miR-3928 in a panel of GBM patient-derived tissue samples (*n* = 17), as well as in commonly used GBM cell lines (*n* = 7; A172, LN18, SNB19, T98G, U87, U251, and U1242). The tumor samples were Grade IV, and post chemo/radio therapies. We found that miR-3928 was down-regulated in the different tissues as well as cell lines that we tested (Figure 1). This finding is consistent with our hypothesis that miR-3928 is a tumor suppressive miRNA.

### 2.2. miR-3928 Inhibits GBM Cell Proliferation and Invasion

**Figure 2:**
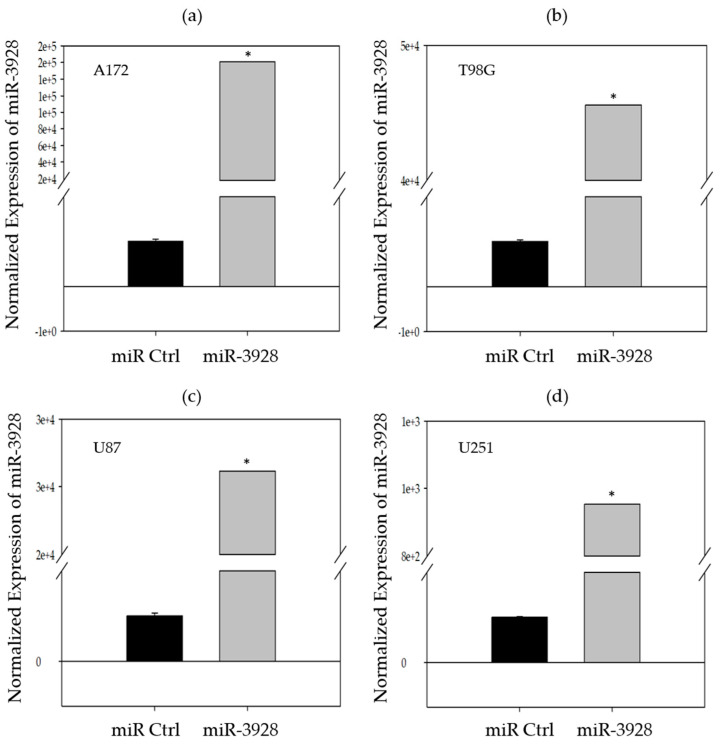
**Confirmation of miR-3928 overexpression in GBM cell lines.** MiR-3928 levels are measured using RT-qPCR and the values are normalized to the same cell lines transfected with microRNA scrambled control and the housekeeping gene, RNU6B, U6B small nuclear RNA gene. (**a**) Exogenous overexpression of transiently transfected miR-3928 in A172 cells. (**b**) Exogenous overexpression of transiently transfected miR-3928 in T98G cells. (**c**) Exogenous overexpression of transiently transfected miR-3928 in U87 cells. (**d**) Exogenous overexpression of transiently transfected miR-3928 in U251 cells. All RT-qPCR samples are subsequently analyzed on an agarose gel to confirm binding specificity. Student’s *t*-tests were performed to compare the qPCR cycle numbers. * = *p* ≤ 0.05.

Because miR-3928 is endogenously downregulated, we overexpressed this miRNA, or a miRNA scrambled control in four different commonly used GBM cell lines (A172, T98G, U87, and U251) to assess its in vitro functional phenotype. As we determined in the previous section, all of these GBM cell lines have low endogenous expression levels of miR-3928. The confirmation of overexpression of miR-3928 is shown in Figure 2 and measured using RT-qPCR.

**Figure 3:**
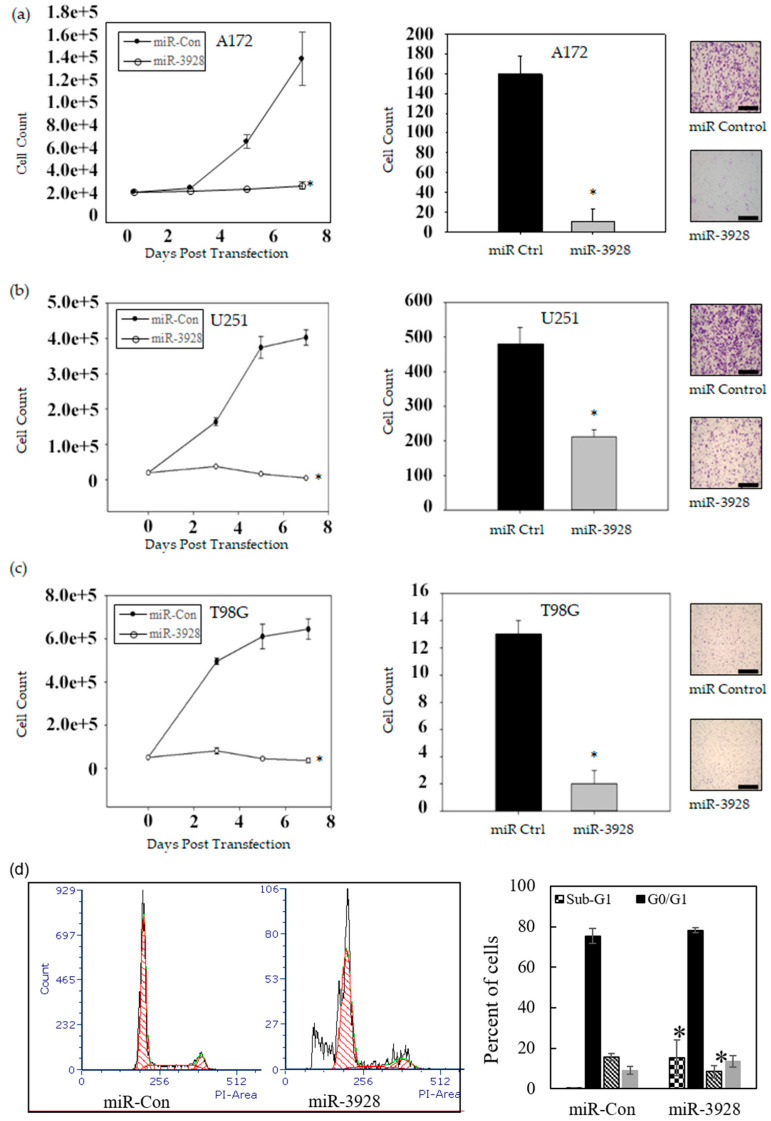
**miR-3928 inhibits GBM cell proliferation and invasion.** (**a**) A172 cells transfected with either miRNA scrambled control or miR-3928 were trypsinized and counted at 3, 5, and 7 days post transfection. Overexpression of miR-3928 inhibits cell growth of A172 cells (left panel); A172 cells transfected with miRNA scrambled control or miR-3928 were also cultured on collagen IV coated Boyden Chambers at 3 days post transfection. Invasive cells were imaged and analyzed. Representative images are shown (right panel). The quantified data show that miR-3928 inhibits cell invasion across the collagen matrix (middle panel). (**b**,**c**) Cell growth and invasion assay in U251 (**b**) and T98G cells (**c**) show that miR-3928 overexpression leads to the inhibition of both cell growth and invasion as compared to scrambled control. All growth assays were performed in triplicates. Scale bars are 1 mm; (**d**) Propidium iodide flow-cytometric cell-cycle analysis shows that miR-3928 increases the sub-G1 population and leads cell cycle arrest at G1/S in A172 cells. * = *p* ≤ 0.05.

Following exogenous transient transfection of either miR-3928 or miRNA scrambled control, in vitro functional assays were performed. The results are shown in Figure 3 and Supplementary Figure S1.

We assessed the effects of miR-3928 on cell growth in GBM cells. The cells were transfected with miR-3928 or scrambled control and counted every two days for seven days. miR-3928 significantly inhibited the cell growth of A172, T98G, U251, and U87 (Left panels of Figure 3a–c and Supplementary Figure S1).

We assessed the effects of miR-3928 on GBM cell invasion. MiR-3928 or miRNA scrambled control were transfected into GBM cells followed by invasion assays. miR-3928 decreased the invasion of GBM cells (middle and right panels of Figure 3a–c).

We also performed cycle analyses by propidium iodide flow cytometry to determine the miR-3928 effects on cell cycle in A172 cells. miR-3928 overexpression strongly increased the sub-G1 population, which marks apoptotic cells, and induced G1/S arrest (Figure 3d).

Taken together, these in vitro functional assays, conducted in multiple GBM cell lines with consistent results, show that miR-3928 is a strong inhibitor of GBM cell growth, survival, and invasion.

### 2.3. miR-3928 inhibits In Vivo GBM Xenograft Growth

**Figure 4:**
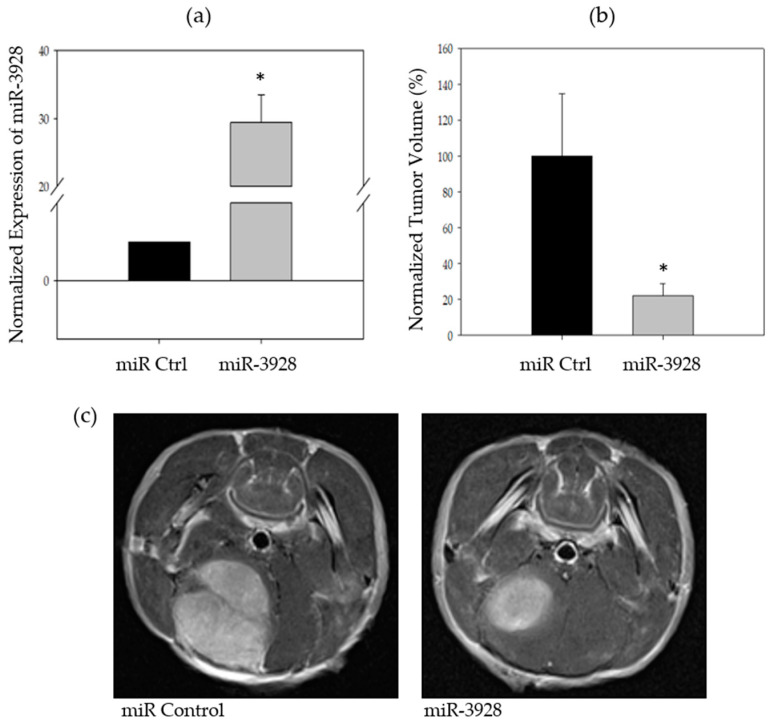
**MiR-3928 inhibits in vivo GBM xenograft growth.** (**a**) MiR-3928 was stably transfected ex vivo in U87 cell lines. Overexpression of exogenously transfected miR-3928 was confirmed with RT-qPCR. (**b**) A cohort of immunocompromised nude mice were stereotactically implanted with U87 GBM cells expressing either miR-3928 or miRNA scrambled control. The mice were imaged with magnetic resonance imaging (MRI) 16 days post injection. Tumor volumes were measured and averaged. MiR-3928 significantly inhibited tumor growth. (**c**) Representative MRI images of miRNA scrambled control and miR-3928 transfected U87 xenografts. * = *p* ≤ 0.05.

We assessed the tumor suppressive capabilities of miR-3928 in an in vivo animal model using immunocompromised nude mice. miR-3928 or miRNA scrambled control was cloned into a lentiviral plasmid and then stably expressed in U87 cells. Overexpression of miR-3928 was confirmed with qPCR (Figure 4a). Stably transfected U87 cells were stereotactically implanted into nude mice brains (*n* = 5 in each experimental group). The animals were monitored for the formation of tumor xenografts. Magnetic resonance imaging (MRI) was performed 16 days post injection. The tumors were quantified and the volumes were calculated and averaged (Figure 4b,c). miR-3928 significantly reduced the tumor volume of U87 xenografts in nude mice as compared to the miRNA scrambled control.

These experiments support the tumor suppressive capabilities of miR-3928 in an in vivo context.

### 2.4. miR-3298 Inhibits the Expressions of Several Oncogenes, including MDM2 and CD44, Leading to Upregulation and Activation of p53 in GBM Cells

Sequence alignments and bioinformatics analyses predicted that miR-3928 can potentially target many important oncogenes in GBM (Figure S4). Our functional studies have shown that miR-3928 has strong tumor suppressive properties in vitro and in vivo. We next investigated the effect of miR-3928 overexpression on the protein expressions of the oncogenes MDM2, CD44, DDX3X, HMGA2, CCND1, BRAF, ATOH8, and BMI1 with immunoblotting.

**Figure 5:**
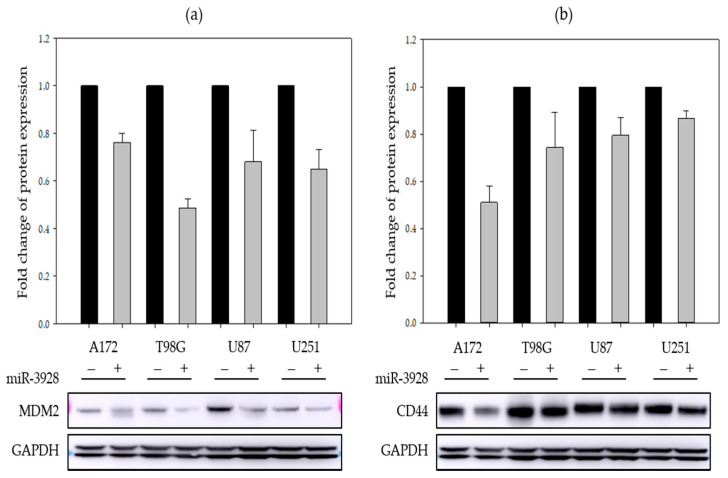
**MiR-3298 inhibits the expressions of MDM2 and CD44 in GBM cells.** (**a**) Band intensities from immunoblot replicates are quantified and graphed for A172, T98G, U87, and U251 cells transfected with either miRNA scrambled control (−) or miR-3928 (+). Representative immunoblot images for MDM2 and associated GAPDH are shown below the bar graph. (**b**) Band intensities from immunoblot replicates are quantified and graphed for A172, T98G, U87, and U251 cells transfected with either miRNA scrambled control (−) or miR-3928 (+). Representative immunoblot images for CD44 and associated GAPDH are shown below the bar graph. For each cell line, the miR-3928 average band intensities are normalized to the miRNA scrambled control band intensities.

GBM cell lines (A172, T98G, U87, and U251) were transfected with either miR-3928 or miRNA scrambled control. Total protein lysate was collected 48 hpost transfection and subjected to immunoblotting for the oncoproteins. We first investigated MDM2 and CD44 protein expressions post miR-3928 transfection. All four different GBM cell lines showed significant inhibition of MDM2 and CD44 protein upon miR-3928 overexpression as compared to the miRNA scrambled control (Figure 5 and Figure S2).

**Figure 6:**
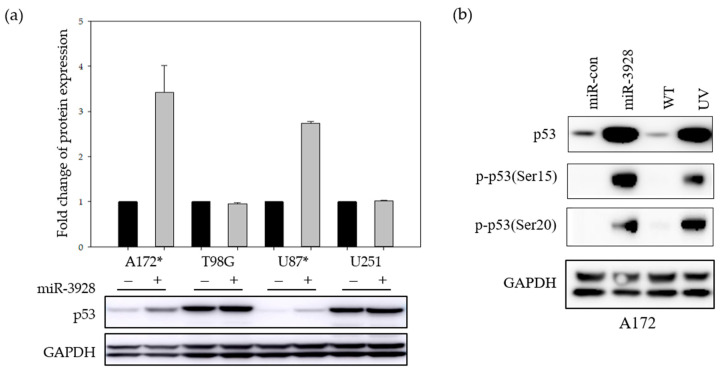
**miR-3928 induces the expression and activation of p53 in GBM cells.** (**a**) Band intensities from immunoblot replicates are quantified and graphed for A172, T98G, U87, and U251 cells transfected with either miRNA scrambled control (−) or miR-3928 (+). Representative immunoblot images for p53 and associated GAPDH are shown below the bar graph. Exogenously overexpressed miR-3928 leads to the upregulation of wild-type p53 in GBM cell lines A172 and U87 that have wild-type p53 (*) but not in T98G and U251 cell lines, which have mutant p53. (**b**) With immunoblots showing that miR-3928 overexpression induces p53 phosphorylation in A172 cells, UV treated cells are used as positive control for p53 phosphorylation/activation.

Because MDM2 is an important regulator of p53, the canonical guardian of the genome tumor suppressor gene [29], we assessed the effects of miR-3928 on the expression and activation of p53. GBM cells were transfected with miR-3928 or scrambled control and p53 expression and activation were determined by immunoblotting for total and phospho-p53. Overexpression of miR-3928 in GBM cell lines with wild-type p53 (A172 and U87) led to an upregulation of p53 (Figure 6a). This was not observed in GBM cell lines with mutant p53 (T98G and U251). Importantly, we also found that overexpression of miR-3928 significantly induces p53 phosphorylation at ser15 and ser20 sites in A172 cells, with UV treated cells used as positive controls (Figure 6b).

**Figure 7:**
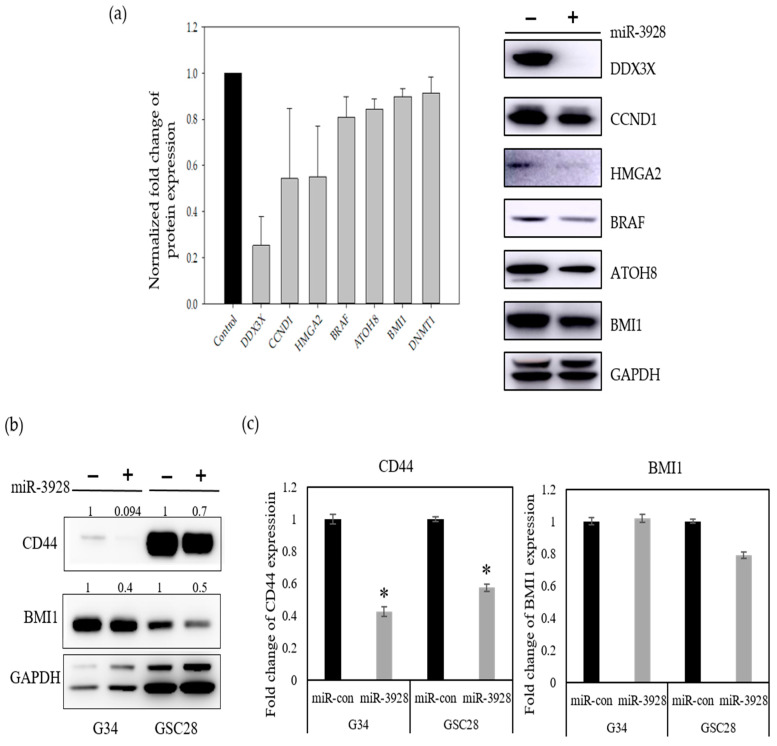
**miR-3928 inhibits the expression of various oncogenes.** (**a**) GBM cell lines A172 and U87 were transfected with either miR-3928 or miRNA scrambled control. Cells were lysed and subjected to immunoblotting for oncogenic proteins predicted to be targeted by miR-3928. Immunoblot band intensities were quantified and averaged. (**b**) GSCs were transfected with miR-3928 or scrambled controls and subjected to immunoblotting for CD44 and BMI1. (**c**) GSCs were transfected with miR-3928 or scrambled controls and CD44 and BMI1 mRNA levels were measured with qRT-PCR normalized to 18sr in the same cells. * = *p* < 0.05.

We also assessed the effects of miR-3928 on the expressions of six additional oncoproteins: DDX3X, CCND1, HMGA2, BRAF, ATOH8, and BMI1 by immunoblotting. Similar to MDM2 and CD44, exogenous overexpression of miR-3928 led to the downregulation of all six oncoproteins in the GBM cell lines tested (A172 and U87) (Figure 7a). Since CD44 and BMI1 are well known cancer stem cell markers, we also investigated the effect of miR-3928 on GBM stem cells (GSCs). We found that miR-3928 reduces CD44 and BMI1 protein expression in GSC G34 and GSC28 (Figure 7b). We also assessed the effects of miR-3928 on the mRNA expression of CD44 and BMI1 by real time qRT-PCR. miR-3928 significantly downregulated CD44 mRNA expression in both G34 and GSC28 but has no effect on BMI1 mRNA expression (Figure 7c). Since miRNAs regulate gene expression by either degrading mRNA or inhibiting protein translation, this suggests that miR-3928 inhibits BMI1 at the protein translation level.

The above data show that miR-3928 inhibits many important oncogenes that regulate a wide range of cancer promoting processes [30,31,32,33,34,35,36,37].

## 3. Discussion

In this study, we investigated the role of miR-3928 in GBM. We demonstrate, for the first time, that miR-3928 is a strong inhibitor of tumor growth and invasion in GBM. We show that miR-3928 is downregulated in GBM tissue and GBM cell lines. We demonstrate that miR-3928 strongly inhibits GBM cell and tumor growth and other malignancy parameters. Therefore, we categorize miR-3928 as a new tumor suppressor miRNA in GBM.

The potential mechanism of miR-3928 function is summarized in Figure S3, from the context of the hallmarks of cancer. miR-3928 acts via the inhibition of several oncogenes and upregulation and activation of p53.

There follows a brief summary of the role the oncogene targets of miR-3928 play in the regulation of cancer. MDM2, or mouse double minute 2 homolog, is a negative regulator of p53, arguably one of the most important tumor suppressors in human cancer. MDM2 exhibits oncogenic properties in GBM due to amplification and overexpression [34]. CD44 is a well-studied protein involved in the differentiation of stem cells. It is a membrane bound adhesion receptor that is involved in the progression and metastasis of cancer cells [38] and is overexpressed in GBM [35]. DDX3X, the DEAD (Asp-Glu-Ala-Asp) box helicase 3, X-linked, is involved in various aspects of tumor migration and proliferation [30]. It has been shown to correlate with poor survival in gliomas. HMGA2, or High Mobility Group At-hook 2, is a transcriptional modulator that plays an important role in normal cell physiology as well as cancer stem cell physiology [32]. CCND1, or cyclin D1, is an important regulator of cell cycle. It is frequently amplified in other cancers such as breast [39] and small-cell B-non-Hodgkin lymphomas [40]. BRAF, or v-raf murine sarcoma viral oncogene homolog B, is well-characterized in a variety of cancers to be a driver of cancer progression [41]. It is also frequently altered in brain tumors. ATOH8, or atonal homolog 8, has been shown to be both tumor suppressive [42] and oncogenic [43] in other cancers, but has not been investigated in glioblastoma. It is involved in the process of embryogenesis and tissue development. BMI1, or B lymphoma Mo-MLV insertion region 1 homolog, has oncogenic properties through the mediation of p16 and p19, important cell cycle genes. In GBM, it helps maintain stem cell renewal [37]. This target is significant in a translational context because it might help circumvent the recurrent nature of GBM because a main driver of GBM recurrence is the presence of GBM stem cells which are resistant against chemo- and radiotherapy [44].

In cell lines with wild-type p53, the degradation of MDM2 also leads to the upregulation and activation of p53, which contributes to the tumor suppressive phenotype. In cell lines with mutant p53, while p53 is not upregulated, the tumor suppressive phenotype is not significantly diminished, probably due to the inhibition of oncogenes other than MDM2. The p53 signaling pathway is altered in 87% of GBM tumors, with MDM2 being amplified in 14% of GBM tumors [45]. Two other oncogene targets worth noting are CD44 and BMI1. Both are involved the maintenance and regulation of GBM stem cells [37,46]. GBM stem cells are arguably responsible for GBM recurrence and resistance to therapy [44,47].

Future translational work will focus on using miR-3928 as an inhibitor in the treatment of GBM. Because of the complex nature of GBM, this work has translational potential since miR-3928 is capable of disrupting multiple signaling pathways, resulting in the degradation of many important oncoproteins and the upregulation of wild-type p53. Since miR-3928 upregulates wild type p53, it might also enhance the radio/chemo sensitivity and can be a potential sensitizing agent for GBM radiotherapy.

In summary, we categorize miR-3928 as a master regulatory miRNA in GBM because it is capable of strongly inhibiting GBM cell growth and invasion by acting on many important oncogenes, as well as on p53.

## 4. Materials and Methods

### 4.1. Cell Lines and Tumor Specimens

Glioblastoma patient tissues were obtained from the University of Virginia Bio-repository and Tissue Specimen Facility according to procedures that were reviewed and approved by the Institutional Review Board. GBM cell lines A172, LN18, SNB19, T98G, U87, U251, and U1241 were purchased from ATCC (Manassas, VA, USA). GBM stem cells GSC28 was a kind gift from Drs. Erik P. Sulman and Krishna Bhat, MD Anderson Cancer Center. GSC G34 was a kind gift from Dr. Jakub Godlewski, Harvard Medical School. The GSCs were derived from patient surgical specimens and characterized for in vivo tumorigenesis, pluripotency, self-renewal, stem-cell markers, and neurosphere formation. A172, LN18, and SNB19 cells were grown in DMEM with 4.5 g/L glucose supplemented with 10% FBS; T98G cells were grown in MEM supplemented with 10% FBS; U87 cells were grown in MEM supplemented with 1 mM sodium pyruvate, 0.15% (*w*/*v*) sodium bicarbonate and 1% non-essential amino acids and 10% FBS; U251 cells were grown in RPMI with 5% FBS; U1242 cells were grown in alpha MEM with L-Glutamine supplemented with 10% FBS. GSCs were grown in Neurobasal Media, L-glutamine (0.5 mM), N2 and B27 supplements (0.5X), and human recombinant bFGF and EGF (50 ng/mL) (R&D Systems, Minneapolis, MN, USA). All the other reagents and media are from Thermo Fisher Scientifics (Waltham, MA, USA). All cells were cultured in media in a 37 °C incubator with 5% CO_2_ and 20% O_2_.

### 4.2. Reagents

The Lipofectamine RNAiMAX transfection reagent and Opti-MEM I Reduced Serum Media (ThermoFisher Scientific, Waltham, MA, USA) were used for all miRNA transfections. Pre-miR-3928, pre-miR, and scrambled control were purchased from Thermo Fisher Scientific (Waltham, MA, USA). Propidium iodide was from BD Pharmingen (San Diego, CA, USA).

### 4.3. Vectors

Lenti-vectors containing miR-3928 or scrambled control sequences and packaging vectors were obtained from System Biosciences (Palo Alto, CA, USA).

### 4.4. Quantitative RT-PCR

The tissue samples were first homogenized. Then, along with cell lines, all samples were lysed with TRIzol. Total miRNA was extracted from each sample using the miRNeasy kit (Qiagen, Chatsworth, CA, USA, Cat. No. 217084). Subsequently, cDNA was synthesized using the miScript II RT Kit (Qiagen, Cat. No. 218161). Quantitative PCR was performed using company designed primer assays for miR-3928 and U6B (housekeeping gene) with the miScript SYBR Green PCR Kit (Qiagen, Cat. No. 218075). The conditions for the qPCR were as described in the kit protocol. Expression analysis was performed as described previously [4]. 100 ng of cDNA was added to each PCR reaction. The specificity of the primer assays was verified through an 1% agarose gel. qRT-PCR primers for CD44, BMI1, and 18sr are: CD44-F, TTTGCATTGCAGTCAACAGTC; CD44-R, GTTACACCCCAATCTTCATGTCCAC; BMI1-F, AATCTAAGGAGGAGGTGA; BMI1-R, CAAACAAGAAGAGGTGGA; 18sr-F: CGGCTACCACATCCAAGGAA, 18sr-R: GCTGGAATTACCGCGGCT.

### 4.5. Cell Growth Assay

GBM cell lines were seeded at densities of 15,000–20,000 cells per well in 6-well plates and transfected 24 h later. MiR-3928 (30 nM) or miRNA (30 nM) scrambled control were transfected into the cell lines with Lipofectamine RNAiMAX transfection reagent. Prior to transfection, complete growth media was replaced by Opti-MEM I Reduced Serum Media (ThermoFisher Scientific). Four hours after transfection with lipofectamine, the media were replaced with complete growth media. After 48 **h** the cells were trypsinized and counted with a hemocytometer, every two days, over the course of seven days as previously described [48].

### 4.6. Invasion Assay

GBM cell lines were seeded at densities of 300,000 cells per well in a 6-well plate and transfected 24 h later with either miR-3928 or scrambled control. After 72 h, the cells were seeded with at a density of 417 cells/mL in 0.01% FBS media in collagen IV coated chambers. The chambers were incubated in complete media with 10% FBS in a 24-well plate in triplicates. After 8 h of incubation at 37 °C in 5% CO_2_ and 20% O_2_, the chambers were gently rinsed with 1xPBS and stained and fixed with 0.1% crystal violet solution in 20% methanol. After drying at room temperature, the chambers were imaged on Evos XL Core microscope (Life Technologies, Grand Island, NY, USA). The images were analyzed with ImageJ Software (National Institutes of Health, Bethesda, MD, USA).

### 4.7. Cell Cycle Assay

GBM cells were seeded at 200,000 cells per well for 24 h. The cells were transfected with miRNA for 72 h as mentioned in cell growth assay. Cells were harvested and fixed in cold 80% ethanol and fixed for 2 h at 4 °C. The cells were washed with PBS and treated by RNase A at 100 µg/mL (ThermoFisher Scientific) and stained with Propidium Iodide at 20 µg/mL (ThermoFisher Scientific). Cells were assayed for DNA content with a FACS Calibur flow cytometer (BD Biosciences, San Jose, CA, USA). The Cell Quest software (BD Biosciences) was used for data analysis.

### 4.8. Animal Experiments

U87 cells were infected with lenti-viral vectors encoding pre-miR-3928 or miR-control. Following antibiotic selection to ensure stable transfection, the cells (2  ×  10^5^) were stereotactically implanted into the striata of immunodeficient mice (*n*  =  5 for each group). Sixteen days after tumor implantation, the brains were scanned with magnetic resonance imaging on a 7 Tesla Bruker/Siemens ClinScan small animal MRI. Tumor volumes were quantified according to previously established and validated protocols [48].

### 4.9. Immunoblot Analysis

GSCs and GBM cell lines were transfected with either miR-3928 or scrambled control using RNAiMAX. Total cell lysate was extracted from each sample 48 h after transfection. The protein concentrations were quantified using the Bradford protein assay according to established protocols. Immunoblots were performed according to standard protocols. Antibodies for the following proteins were used: p53 (sc-263; Santa Cruz Biotechnology, Inc., Santa Cruz, CA, USA), MDM2 (OP115; Cell Signaling Technology), CD44 (3570S, Cell Signaling Technology, Danvers, MA, USA), DDX3X (sc-81247, Santa Cruz Biotechnology), HMGA2 (8179S; Cell Signaling Technology), CCND1 (sc-8396, Santa Cruz Biotechnology), BRAF (sc-5284, Santa Cruz Biotechnology), ATOH8 (PA5-2710, Thermo Fisher Scientific), BMI1 (6964S, Cell Signaling Technology), p-p53(Ser15) (9286S, Cell Signaling Technology), and p-p53(Ser20) (9287S, Cell Signaling Technology). Every immunoblot was also immunostained with GAPDH (Santa Cruz Biotechnology) as a loading control. All antibodies were used at a dilution of 1:500.

### 4.10. Statistical Analysis

All experiments were performed in at least three replicates. Where appropriate, two group comparisons were analyzed with a Student’s *t*-test, and the *p*-values were calculated.

## Data Availability

Not applicable.

## Supplementary Materials

The following are available online at https://www.mdpi.com/article/10.3390/ijms23073930/s1.
