# Supplementary material for: MicroRNA 3928 Suppresses Glioblastoma through Downregulation of Several Oncogenes and Upregulation of p53
Source: Int J Mol Sci. 2022 Apr 1;23(7):3930. doi: 10.3390/ijms23073930 (PMC8998958; doi:10.3390/ijms23073930)
Supplement: Supplementary file 1 [file ijms-23-03930-s001.zip › ijms-1500248-supplementary.pdf]

International Journal of
Molecular Sciences

MDPI

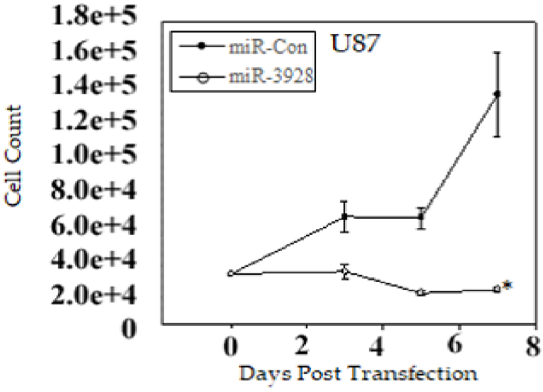

Figure S1. miR-3928 effect on GBM cell proliferation. U87 cells transfected with either miRNA scrambled control or miR-3928 were collected and counted at 3, 5, and 7 days' post transfection. Overexpression of miR-3928 inhibits growth of U87 cells.

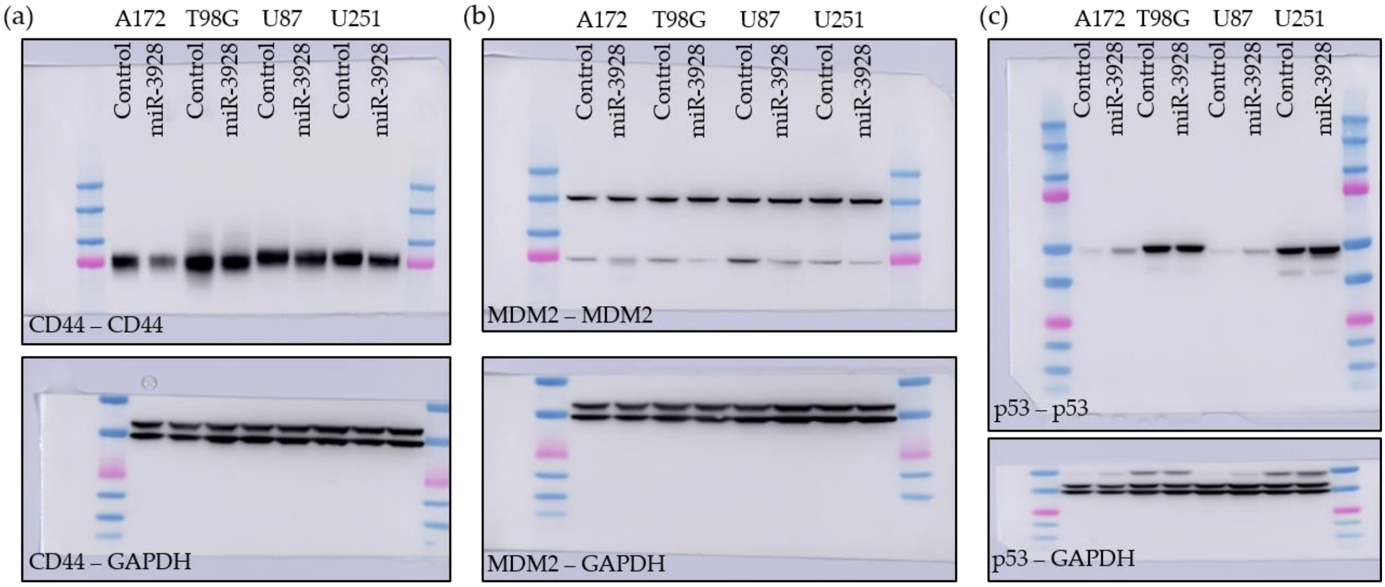

Figure S2. Representative raw immunoblots. (a) Immunoblot of CD44 and associated GAPDH from cell lysates collected from A172, T98G, U87, and U251 cells that were transiently transfected with either microRNA scrambled control (Control) or miR-3928. (b) 3 Immunoblot of MDM2 and associated GAPDH from cell lysates collected from A172, T98G, U87, and U251 cells that were transiently 4 transfected with either microRNA scrambled control (Control) or miR-3928. (c) Immunoblot of p53 and associated GAPDH from cell 5 lysates collected from A172, T98G, U87, and U251 cells that were transiently transfected with either microRNA scrambled control 6 (Control) or miR-3928. All immunoblots were performed in 3 replicates.

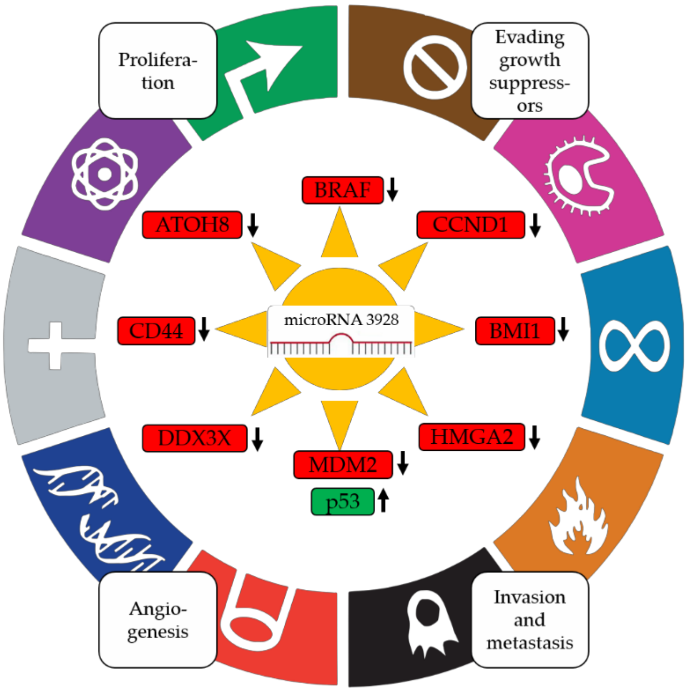

Figure S3. Master regulatory miR-3928 and the hallmarks of cancer [Hanahan, D. and R.A. Weinberg, *Hallmarks of cancer: the next generation*. Cell, 2011. **144**(5): p. 646-74.]. MiR-3928 acts on GBM cells in vitro and in vivo through the downregulation of important oncogenes (red) and, by way of MDM2, through the upregulation of the important tumor suppressor protein, p53 (green). These proteins are involved in various cancer-promoting pathways such as proliferation, evading growth suppressors, angiogenesis, invasion and metastasis.

```
cluster-ATOH8            TAATTTACAATAACCACCAGGATTCCTCCG  30
miR-3928-revcomp27       ----------------------TTCCTC--  6
                                               ******

cluster-BMI1             TCTCCTCATCCACAGTTTCCTCACATTTCCAGTACTATG 39
miR-3928-revcomp27       ----------------TTCCTC----------------- 6
                                         ******

cluster-BRAF             TTACATCTTCTTCCTCTTCTAGCCTTTCAG  30
miR-3928-revcomp27       ----------TTCCTC--------------  6
                                   ******

cluster-CCND1-1          TTGAACACTTCCTCTCCAAAATG 23
miR-3928-revcomp27       --------TTCCTC--------- 6
                                 ******

cluster-CCND1-2          CCCCAACAACTTCCTGTCCTACTACCGCCTCACACGCTTCCTCTCCAG        48
miR-3928-revcomp27       -------------------------------------TTCCTC-----        6
                                                              ******

cluster-CD44             TTATTGTTACTTTGACTTTTCAGAGCACACCCTTCCTCTGGTTTTTGTATATTTATTGAT    60
miR-3928-revcomp27       --------------------------------TTCCTC----------------------    6
                                                         ******

cluster-DDX3X            CATACATTCAAAGCACTGTTTTCAAAGTTAATGCAAGTAAATACAGCAATTCCTCTTTCA    60
miR-3928-revcomp27       -------------------------------------------------TTCCTC-----    6
                                                                          ******

cluster-HMGA2            GATAATTTTCCTCAATCACACTACACATCACACAAG    36
miR-3928-revcomp27       -------TTCCTC-----------------------    6
                                ******

cluster-MDM2             ACAGGGTCAGCATGTGGAATTCCAAGATACCTCTTGACTTCCTCTCAAGCTCCGTGTTTG    60
miR-3928-revcomp27       --------------------------------------TTCCTC----------------    6
                                                               ******
```

Figure S4. The miR-3928 seed sequence alignment to each target gene. The cluster alignment based on Target Scan (online) and our PAR-CLIP analysis (unpublished).
